# Plasma phosphorylated-tau217 is increased in Niemann–Pick disease type C

**DOI:** 10.1093/braincomms/fcae375

**Published:** 2024-10-25

**Authors:** Fernando Gonzalez-Ortiz, Thomas K Karikari, Danielle Taylor-Te Vruchte, Dawn Shepherd, Bjørn-Eivind Kirsebom, Tormod Fladby, Frances Platt, Kaj Blennow

**Affiliations:** Department of Psychiatry and Neurochemistry, Institute of Neuroscience and Physiology, the Sahlgrenska Academy at the University of Gothenburg, Mölndal, 43180, Sweden; Clinical Neurochemistry Laboratory, Sahlgrenska University Hospital, Mölndal, 43180, Sweden; Department of Psychiatry and Neurochemistry, Institute of Neuroscience and Physiology, the Sahlgrenska Academy at the University of Gothenburg, Mölndal, 43180, Sweden; Department of Psychiatry, School of Medicine, University of Pittsburgh, Pittsburgh, PA 15203, USA; Department of Pharmacology, University of Oxford, Oxford OX1 3QT, UK; Department of Pharmacology, University of Oxford, Oxford OX1 3QT, UK; Department of Neurology, University Hospital of North Norway, Tromsø, 9019, Norway; Department of Psychology, Faculty of Health Sciences, The Arctic University of Norway, Tromsø, 9031, Norway; Department of Neurology, Akershus University Hospital, Lørenskog, 1478, Norway; Institute of Clinical Medicine, Campus Ahus, University of Oslo, Oslo, 0316, Norway; Department of Pharmacology, University of Oxford, Oxford OX1 3QT, UK; Department of Psychiatry and Neurochemistry, Institute of Neuroscience and Physiology, the Sahlgrenska Academy at the University of Gothenburg, Mölndal, 43180, Sweden; Clinical Neurochemistry Laboratory, Sahlgrenska University Hospital, Mölndal, 43180, Sweden; Institut du Cerveau et de la Moelle épinière (ICM), Pitié-Salpêtrière Hospital, Sorbonne Université, Paris, 75013, France; University of Science and Technology of China, Hefei, Anhui, 230026, China

**Keywords:** tau pathology, blood biomarkers, lysosomal dysfunction, dementia, neurodegeneration

## Abstract

Niemann–Pick disease type C and Alzheimer’s disease are distinct neurodegenerative disorders that share the presence of neurofibrillary tangle pathology. In this multicentre study, we measured plasma phosphorylated-tau217 in controls (*n* = 60), Niemann–Pick disease type C (*n* = 71) and Alzheimer’s disease (*n* = 30 positive for amyloid and negative for tau in CSF [A^+^T^−^] and *n* = 30 positive for both [A^+^T^+^]). Annual Severity Increment Score and Lysotracker measurements were evaluated in the Niemann–Pick disease type C group to estimate the rate of progression and lysosomal enlargement, respectively. In the cross-sectional analysis, plasma phosphorylated-tau217 was increased in Niemann–Pick disease type C compared with controls (2.52 ± 1.93 versus 1.02 ± 0.34 pg/mL, respectively, *P* < 0.001) and inversely correlated with age at disease onset (*R* = −0.54, *P* < 0.001). In the longitudinal analysis, plasma phosphorylated-tau217 was associated with disease progression determined by Annual Severity Increment Score (*R* = 0.48, *P* < 0.001) and lysosomal enlargement (*R* = 0.26, *P* = 0.004). We found no differences between A^+^T^−^ Alzheimer’s disease and Niemann–Pick disease type C (2.67 ± 1.18 versus 2.52 ± 1. 93 pg/mL, *P* = 0.31); however, A^+^T^+^ Alzheimer’s disease had significantly higher levels than Niemann–Pick disease type C (3.26 ± 1.36 versus 2.52 ± 1.93 pg/mL, *P* = 0.001). Our findings suggest that plasma p-tau217 can increase in brain disorders with isolated tau pathology. Plasma p-tau217 associations with disease progression and severity make it a potential marker in Niemann–Pick disease type C.

## Teaser

Gonzalez-Ortiz *et al*. report the association of plasma phosphorylated-tau217 (p-tau217) with disease severity in Niemann–Pick disease type C and compared levels with healthy controls and Alzheimer’s disease. Concentrations of plasma p-tau217 in Niemann–Pick disease type C were similar to A^+^T^−^ Alzheimer’s disease, but lower than A^+^T^+^ Alzheimer’s disease.

## Graphical Abstract

**Graphical Abstract:**
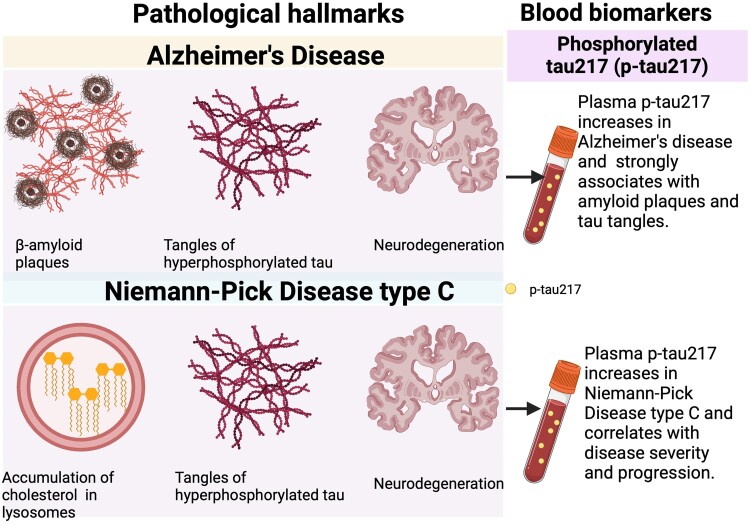

## Introduction

Niemann–Pick disease type C and Alzheimer’s disease are distinct neurodegenerative disorders with different clinical manifestations, age at onset, aetiology and pathogenesis.^1,2^ Niemann–Pick disease type C is primarily characterized by the accumulation of cholesterol and sphingolipids within late endocytic compartments, leading to neurodegeneration and cognitive impairment.^3^ Unlike Alzheimer’s disease, which is characterized by the presence of amyloid plaques primarily composed of amyloid β (Aβ),^4,5^ Niemann–Pick disease type C does not exhibit this pathological feature.^6^ While the dysregulation of amyloid metabolism may be present in patients with Niemann–Pick disease type C, amyloid plaque formation has not been observed in *in vivo* or *in vitro* studies.^2,7,8^ However, Niemann–Pick disease type C and Alzheimer’s disease present some remarkable similarities at the molecular and anatomopathological levels,^1-3^ specifically the presence of neurofibrillary tangles (NFTs) composed of hyperphosphorylated tau.^4-6,9^ Moreover, neuropathological studies suggest that NFTs in Alzheimer’s disease and Niemann–Pick disease type C show similar patterns of phosphorylation on some key epitopes (e.g. T181, S202, T205, T231, S262 and S396).^2,10^

In recent years, plasma phosphorylated tau (p-tau) species, particularly p-tau217, have emerged as promising candidates for detecting Alzheimer’s disease pathology.^4,11-13^ Multiple studies have reported strong correlations between elevated levels of plasma p-tau217 and the presence and severity of both Aβ deposition and tau pathology in the brain assessed by PET,^11,14,15^ as well as at follow-up autopsy examination.^16^ These findings suggest a potential relationship between plasma p-tau markers and the amounts and extent of Alzheimer’s disease neuropathological changes.^17,18^ Recommendations from the Alzheimer’s Association and other expert groups^19-21^ have all suggested that plasma p-tau217 should be used as a surrogate biomarker for Aβ pathology for clinical applications of blood biomarkers, including for the monitoring of patients on anti-amyloid treatments.^11,14,18,22^ More recently, The National Institute on Aging and the Alzheimer’s Association have included plasma p-tau as a Core 1 biomarker in the revised criteria for Alzheimer’s disease diagnosis,^23^ suggesting that abnormalities in plasma p-tau are equally as significant as CSF biomarker abnormalities or amyloid PET. However, recent reports have suggested that CSF p-tau217 is not only increased in Alzheimer’s disease but also in non-Alzheimer’s disease neurodegenerative tauopathies including those harbouring specific disease-associated genetic mutations in the *MAPT* gene without evidence of Aβ pathology (that is, they have A^−^T^+^ profiles that are outside the Alzheimer’s disease continuum).^24,25^ It is unknown whether plasma p-tau217—a more accessible biomarker than CSF p-tau217—is elevated in A^−^T^+^ tauopathies, such as Niemann–Pick disease type C.

Diagnosis and evaluation of patients with Niemann–Pick disease type C rely on clinical manifestations, family history and genetic analysis.^3,26^ However, easily accessible and reliable blood-based biomarkers that could be used to support diagnosis and monitoring of patients with Niemann–Pick disease type C are lacking.^3,27^ Moreover, understanding the complex interplay between tau pathology in Alzheimer’s disease and Niemann–Pick disease type C offers opportunities to gain new insights into the underlying mechanisms and potential strategies for lysosome and tau-targeting therapies.^1,28-31^

## Materials and methods

### Participant characteristics

The Niemann–Pick disease type C group consisted of 71 patients from 2 independent international clinical cohorts: 36 from the UK (Manchester) and 35 from Germany (Mainz). The control group consisted of 60 participants who were first-degree relatives of the patients with Niemann–Pick disease type C but without evidence of pathology. The age and sex of the control group were not available for analysis. In the current study, longitudinal data for the Niemann–Pick disease type C group were collected. Research on data obtained from patients with Niemann–Pick disease type C was covered by REC/IRB approvals 06/MRE02/85 (UK) and S-032/2012 (Germany). Written informed consent, and if applicable, assent, were obtained in each centre. The Annual Severity Increment Score (ASIS) was evaluated in the Niemann–Pick disease type C group to estimate the rate of progression.^26^ The higher the severity score, the greater the disease burden. Clinical assessments were conducted by different individuals in the two centres. Lysosomal enlargement was assessed used the Lysotracker method previously reported in the Niemann–Pick disease type C group.^32^ Predementia patients with Alzheimer’s disease for the current project were sourced from the Norwegian Dementia Disease Initiation (DDI) cohort database. This is a multicentre longitudinal study recruiting patients at risk for dementia across all of Norway.^33^ The participants sign written informed consent, and the study is approved by the Regional Ethics board (REK 2013/150). Demographic characteristics of the participants are found in Supplementary Tables 1 and 2.

### Sample collection and biomarker measurements

Plasma samples were obtained according to standard procedures and stored at −80°C until use. Plasma p-tau217 and p-tau231 were measured on the Simoa HD-X platform using the University of Gothenburg in-house protocol as previously described.^11,34^ Plasma Neurofilament light (NfL) was measured using Quanterix commercial kits (#103670). Biomarker measurements were performed between October 2022 and July 2023 at the University of Gothenburg, Sweden.

### Statistical analyses

Statistical analyses were performed with Prism version 9.3.1 (GraphPad, San Diego, CA, USA). Data are shown as mean ± SD unless otherwise stated. The distributions of data sets were examined for normality using the Kolmogorov–Smirnov test. Non-parametric tests were used for non-normally distributed data. The Spearman correlation and the χ^2^ test were used for continuous and categorical variables, respectively. Diagnostic performances were evaluated with receiver-operating curves (ROCs) and area under the curve (AUC) assessments. Linear mixed-effects models were employed to assess changes in plasma NfL, p-tau217 and p-tau231 over time. In order to capture potential non-linear changes in the biomarker over time, each model incorporated a basis spline function with 3 degrees of freedom for the time predictor. To visually compare differences in change over time between the biomarkers, the markers were standardized prior to analyses. To adjust for repeated measurements within subjects and variations in follow-up time between subjects, each model was fitted with a random intercept for subject and random slope for time. The estimates for the biomarkers at baseline for healthy controls (HCs) were added to the plot as reference. An additional model was fitted with interaction terms for age groups (2 through 10 years, 11 through 21 years and 22 through 49 years) for ptau-217 (unstandardized). Statistical analyses and plots were carried out using Rstudio (R version 4.2.2) using lme4, ggeffects and ggplot2 packages.

## Results

In this study, we evaluated baseline and longitudinal changes in plasma p-tau217 and its associations with clinical and biochemical features in a multicentre cohort of patients with Niemann–Pick disease type C (*n* = 71). Furthermore, we compared levels of plasma p-tau217 in patients with Niemann–Pick disease type C with HCs (*n* = 60) and patients with Alzheimer’s disease (*n* = 60).

**Figure 1:**
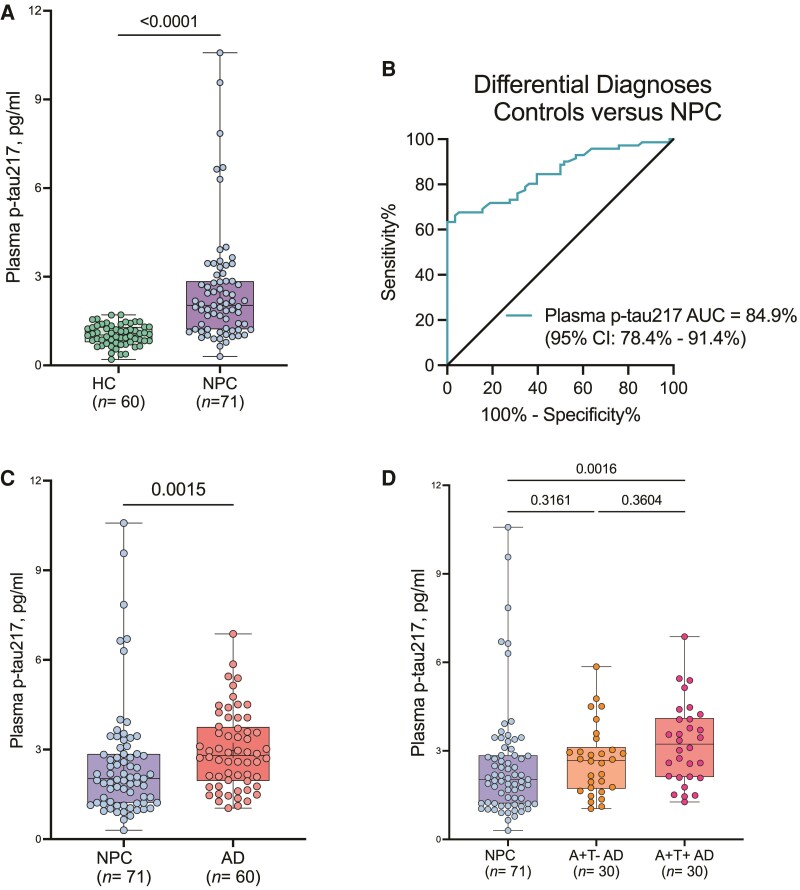
**Comparison of plasma p-tau217 levels in HC, Niemann–Pick disease type C and Alzheimer’s disease.** (A) Concentrations of plasma p-tau217 in HC and Niemann–Pick disease type C showing significant increases in Niemann–Pick disease type C. The corresponding ROC and AUC value indicating between-group (HC versus Niemann–Pick disease type C) discriminatory accuracy of plasma p-tau217 is shown in B. The diagonal line on the ROC curve shows 50% accuracy meaning no difference from chance events. (C) Concentrations of plasma p-tau217 in Niemann–Pick disease type C and the Alzheimer’s disease group. (D) Comparison of plasma p-tau217 in Niemann–Pick disease type C versus CSF amyloid positive (A^+^) tau negative (T^−^) Alzheimer’s disease and A^+^T^+^ Alzheimer’s disease. Group differences were examined using the Mann–Whitney test (two categories) or the Kruskal–Wallis test with Dunn’s multiple comparisons (three groups). In each box plot, the horizontal bar on top of the coloured area shows the 75% percentile, the middle bar depicts the median and the lower bar shows the 25% percentile. Values that are above the 75% percentile and below the 25% percentile are shown outside the coloured area. AD, Alzheimer’s disease; NPC, Niemann–Pick disease type C.

Cross-sectional analysis showed significantly higher levels of plasma p-tau217 in Niemann–Pick disease type C compared with HC (2.52 ± 1.93 versus 1.02 ± 0.34 pg/mL, respectively, *P* < 0.001, Fig. 1A). The fold increase was 1.8, with an AUC of 0.84 (Fig. 1B). Age of diagnosis in the Niemann–Pick disease type C group correlated negatively with the levels of plasma p-tau217 (*R* = −0.54, *P* < 0.001, Supplementary Fig. 1A), suggesting that higher levels of plasma p-tau217 are associated with earlier presentation in more aggressive forms of the disease in agreement with previous reports.^35^ Longitudinal measurements of plasma p-tau217 correlated positively with relative fibroblast lysosomal volumes (*R* = 0.26, *P* = 0.004, Supplementary Fig. 1B), assessed using the LysoTracker method,^32^ and with rates of disease progression using the ASIS (*R* = 0.48, *P* < 0.001, Supplementary Fig. 2A).^26^

**Figure 2:**
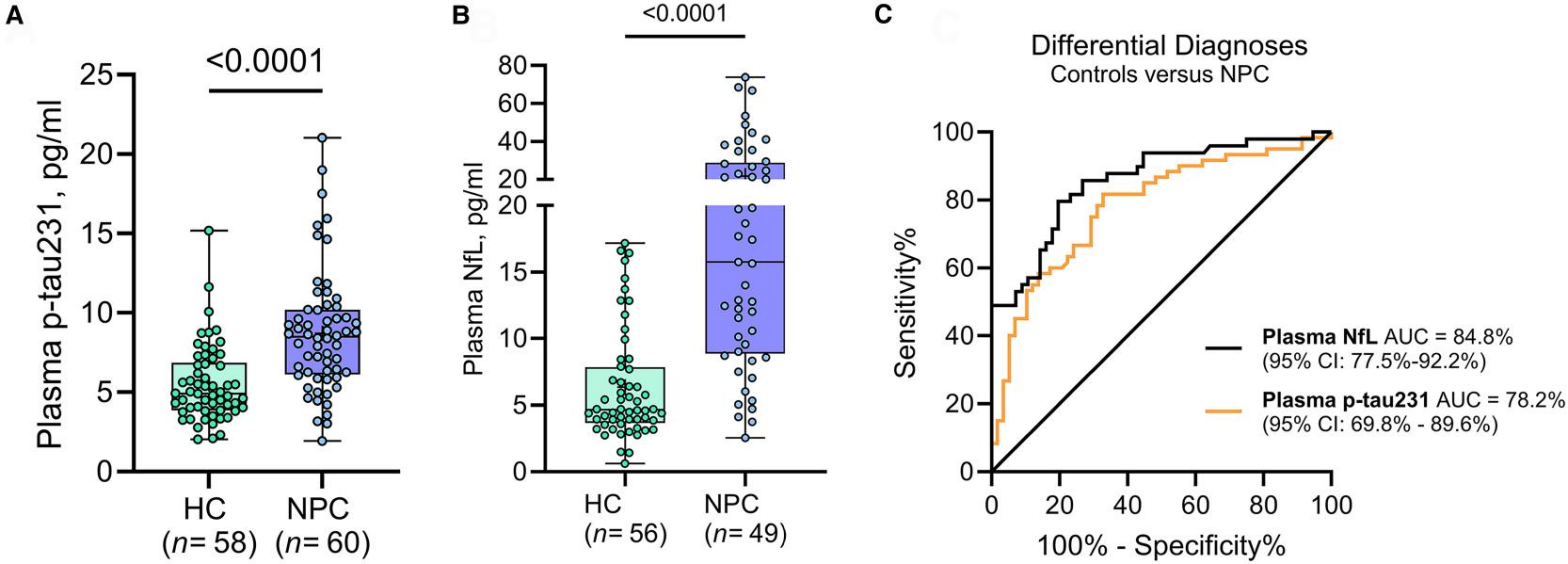
**Plasma p-tau231 and NfL in HC and Niemann–Pick disease type C.** (**A** and **B**) Concentrations of plasma p-tau231 and NfL in HC and Niemann–Pick disease type C showing significant increases for both plasma markers in Niemann–Pick disease type C. In each box plot, the horizontal bar on top of the coloured area shows the 75% percentile, the middle bar depicts the median and the lower bar shows the 25% percentile. Values that are above the 75% percentile and below the 25% percentile are shown outside the coloured area. *P*-values indicate the results of Mann–Whitney tests. The corresponding ROC and AUC values indicating between-group discriminatory accuracies of the biomarkers are shown in (**C**). The diagonal line on the ROC curve shows 50% accuracy meaning no difference from chance events. NPC, Niemann–Pick disease type C.

In addition to plasma p-tau217, we evaluated another high-promising Alzheimer’s disease biomarker, plasma p-tau231^34^ in the Niemann–Pick disease type C (*n* = 60) and HC (58) groups. While plasma p-tau231 was also increased in the Niemann–Pick disease type C group versus HC (8.70 ± 3.85 versus 5.51 ± 2.40 pg/mL, respectively, *P* < 0.0001, Fig. 2A), it showed a less marked increase and was less able to differentiate Niemann–Pick disease type C from HC (AUC: 0.77) when compared with plasma p-tau217. Moreover, the associations of plasma p-tau231 with age of diagnosis (*R* = −0.38, *P* = 0.03, Supplementary Fig. 1C), fibroblast lysosomal volumes (*R* = 0.24, *P* = 0.010, Supplementary Fig. 1D) and ASIS (*R* = 0.45, *P* < 0.0001, Supplementary Fig. 2B) were all weaker than those of plasma p-tau217. These results indicate that plasma p-tau217 is a better indicator of tau pathology and clinical progression in Niemann–Pick disease type C than plasma p-tau231.

NfL in CSF has previously been reported as a potentially useful biomarker in Niemann–Pick disease type C,^36^ reflecting neurodegeneration in the affected patients. In this study, we observed that, similar to recent reports,^37^ plasma NfL was increased in Niemann–Pick disease type C (*n* = 49) compared with HC (*n* = 56) and showed high accuracy differentiating these groups (21.73 ± 17.70 versus 6.34 ± 4.19 pg/mL, respectively, AUC: 0.84, Fig. 2B and C). However, in this sample, we found no significant correlation between plasma NfL and fibroblast lysosomal volumes (Supplementary Fig. 1F) which suggests that plasma NfL is a general marker of neurodegeneration or potential peripheral contamination which makes it less reliable than its CSF version.^38,39^

**Figure 3:**
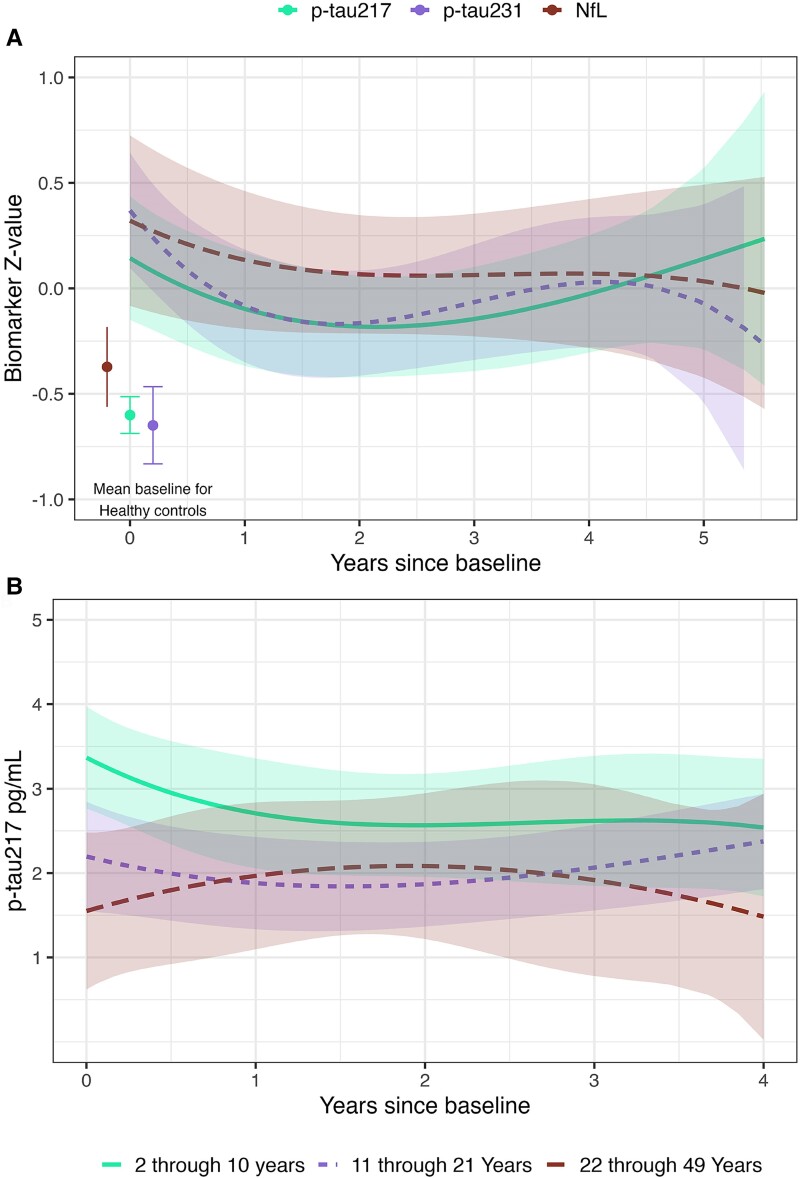
**Longitudinal trajectories of plasma markers in Niemann–Pick disease type C.** (**A**) Curve-linear longitudinal trajectories (basis splines with 3 degrees of freedom) over time of *z*-standardized plasma p-tau217, p-tau231 and NfL in patients with Niemann–Pick disease type C. The coloured bands are 95% CIs of the model estimates for each biomarker. The concentrations for HCs at baseline are shown as individual points, and error bars are 95% CIs of the estimates. (**B**) Curve-linear longitudinal trajectories (basis splines with 3 degrees of freedom) of p-tau217 pg/mL over time in the different age groups. The coloured bands are 95% CIs of the model estimates for each age group.

Longitudinal analysis showed similar trajectories for plasma p-tau231 and NfL (Fig. 3A), with the highest levels at baseline and then a tendency to decrease but without reaching levels of HC. Plasma p-tau217 had a different trajectory (Fig. 3A), with levels showing a first peak at baseline and a second peak at the latest time points (≥5 years). These findings suggest that, unlike plasma p-tau231 or NfL, plasma p-tau217 may be a superior indicator of disease severity and progression in later stages of the disease, in agreement with the results of the cross-sectional analyses. Interestingly, plasma p-tau217 has also been reported as a superior prognostic marker in Alzheimer’s disease when compared with other p-tau species,^13,15,18^ which may suggest that the dynamic of this marker in Niemann–Pick disease type C and Alzheimer’s disease is remarkably similar.

We further divided the Niemann–Pick disease type C participants into three age groups (2−10, 11–21 and 22–49 years old, Fig. 3B) to study the longitudinal trajectories of plasma p-tau217. We observed different patterns in longitudinal trajectories depending on the baseline age group. The youngest group of patients (2–10 years old) had the highest concentrations at baseline, supporting the negative correlation with age, and the levels remained the highest compared with the other age groups. The older age groups showed lower levels of plasma p-tau217 at baseline. The 11- to 21-year-old group exhibited a tendency to increase in levels of plasma p-tau217, while the 22- to 49-year-old group showed a decrease in p-tau217 levels over time (Fig. 3B). Overall, the longitudinal trajectories of plasma p-tau217 in the different age groups seemed to reflect the heterogenous presentation of Niemann–Pick disease type C, with early-life presentations being the most severe form of the disease, and the one with the highest plasma p-tau217 levels, while later age of onset was associated with milder forms of the disease. Agreeably, the correlation of plasma p-tau217 with clinical outcome (ASIS)^26^ and measures of lysosomal dysfunction (Lysotracker staining)^32^ support these findings.

To ascertain how plasma p-tau217 concentrations compared between patients with Niemann–Pick disease type C and Alzheimer’s disease across the disease continuum (early stage with A^+^T^−^ profile and A^+^T^+^ individuals with more established pathology), we compared the levels in patients with Niemann–Pick disease type C at baseline against 60 patients with Alzheimer’s disease determined by CSF biomarker positivity (A^+^, as defined by CSF Aβ42/40 < 0.071 and T^+^ as defined by CSF p-tau >50 pg/mL).^19^ Mean concentrations of plasma p-tau217 in the entire Alzheimer’s disease group were significantly higher than the levels in the Niemann–Pick disease type C group (2.97 ± 1.30 and 2.52 ± 1.93 pg/mL, respectively, *P* = 0.0015, Fig. 1C). We then divided the Alzheimer’s disease group into two categories according to their CSF biomarker profiles, 30 patients with A^+^T^−^ and the remaining 30 with A^+^T^+^ profiles. The A^+^T^−^ Alzheimer’s disease group showed no significant differences in the levels of plasma p-tau217 when compared with the Niemann–Pick disease type C group (2.67 ± 1.18 versus 2.52 ± 1. 93 pg/mL, *P* = 0.31, Fig. 1D). However, the Alzheimer’s disease A^+^T^+^ presented significantly higher levels of plasma p-tau217 compared with Niemann–Pick disease type C (3.26 ± 1.36 versus 2.52 ± 1.93 pg/mL, *P* = 0.001, Fig. 1D). These findings suggest that the concentrations of plasma p-tau217 in patients with Niemann–Pick disease type C were equivalent to those in the early Alzheimer’s disease continuum. In effect, these results show that the Niemann–Pick disease type C and A^+^T^−^ Alzheimer’s disease participants evaluated presented equivalent levels of plasma p-tau217.

## Discussion

An unresolved question in the neurodegenerative disease field is whether increases in plasma p-tau217 are specifically elevated in Alzheimer’s disease or whether high levels can also be observed in other diseases. In this study, we sought to study whether tau pathology by itself results in an increase in plasma p-tau217, or whether Aβ pathology is necessary. This cannot be specifically studied in Alzheimer’s disease, where both tau and Aβ pathology are present, and findings of high plasma p-tau in patients with positive amyloid, but negative tau, PET scans do not preclude the possibility of early stage Alzheimer’s disease where patients have emerging tau pathology, below the threshold for detectability by tau PET.^12^ We therefore examined plasma p-tau217 in patients with Niemann–Pick disease type C, a cholesterol storage disease characterized by tau pathology without amyloid plaque formation.^7,8^ We found that plasma p-tau217 levels were significantly elevated in the patients with Niemann–Pick disease type C relative to unaffected controls and the levels were similar to those in Alzheimer’s disease participants with an A^+^T^−^ profile. These results suggest that tau pathology can drive increases in plasma p-tau217 independently of Aβ pathology.

While plasma p-tau217 was higher in patients with Niemann–Pick disease type C compared with HC, early age at baseline (generally indicative of a more rapidly progressing form of the disease) and the magnitude of lysosomal dysfunction were associated with more marked increases in plasma p-tau217. When the plasma p-tau217 concentrations in Niemann–Pick disease type C were compared with those in the Alzheimer’s disease group, they were not significantly different from the concentrations in patients with A^+^T^−^ profile. These findings suggest that plasma p-tau217 is a marker of disease severity in both Niemann–Pick disease type C and Alzheimer’s disease. However, a key distinction is that in Alzheimer’s disease, the biomarker levels continue to rise when the affected individuals become A^+^T^+^ compared with the A^+^T^−^ participants. This observation is understandable given that Alzheimer’s disease is a mix of two major pathologies—Aβ and tau—while Niemann–Pick disease type C is predominantly tau pathology.

Several studies show that plasma p-tau217 is not increased in non-Alzheimer’s disease neurodegenerative diseases.^16^ Importantly, some of these diseases—such as frontotemporal dementia, progressive supranuclear palsy and corticobasal degeneration—are tauopathies that are known to show accumulation of p-tau at autopsy,^24^ but whether the amounts of NFTs are similar to those in Alzheimer’s disease is unknown. Nonetheless, plasma p-tau217 is not increased in any of these diseases^40^ but its increases were clear in Niemann–Pick disease type C as demonstrated in the present study. These novel results show that the elevations of plasma p-tau217 are selective to specific tauopathies, the reason for which is currently unclear. The high associations with the lysosomal dysfunction measures lead us to hypothesize that further investigations focusing on this link could provide a mechanistic explanation and how that compares with other tauopathies. High levels of plasma p-tau217 in patients with Niemann–Pick disease type C could be a result of lysosomal dysfunction and secondary dysregulation in the metabolism of tau, thus providing an indication of disease severity and progression.

This is the first study showing the potential clinical value of plasma p-tau217 in Niemann–Pick disease type C. High levels of plasma p-tau217 in Niemann–Pick disease type C were associated with disease severity and rapid progression, supporting its use as a potential prognostic/monitoring biomarker in Niemann–Pick disease type C, a disease that currently lacks accessible blood biomarkers. In contrast, plasma p-tau231 and NfL showed weaker associations with clinical outcome or lysosomal dysfunction in Niemann–Pick disease type C, further highlighting the promising utility of plasma p-tau217 in Niemann–Pick disease type C.

Beyond the potential applications in Niemann–Pick disease type C, our findings suggest that plasma p-tau217 is not solely an amyloid dependent marker. While joint amyloid and tau pathology, like in A^+^T^+^ Alzheimer’s disease, seem to have a stronger effect on levels of this marker in plasma, we showed that isolated amyloid pathology (A^+^T^−^ Alzheimer’s disease) and isolated tau pathology (Niemann–Pick disease type C) have a similar effect on levels of p-tau217 in blood, suggesting that the elevation of p-tau217 can be associated with amyloid and tau pathology, and the mechanisms behind these increases could be more in relation to the dysregulation in the shared pathways between amyloid and tau (e.g. lysosomal clearance and lipid metabolism). Understanding how Niemann–Pick disease type C–associated lysosomal dysfunction contributes to tau alterations could unveil novel therapeutic avenues, potentially applicable to Alzheimer’s disease.^30,31^

### Strengths and limitations

The main strength of our study is the inclusion of 60 cases of Niemann–Pick disease type C, a rare neurodegenerative disease, which provides a significant sample size for studying this condition. The longitudinal data collected across these cases further strengthens the study by enabling the assessment of biomarker changes and progression over time in Niemann–Pick disease type C. Additionally, the comparison with a well-characterized Alzheimer’s disease group allows for meaningful comparisons between Niemann–Pick disease type C and Alzheimer’s disease.

Despite its strengths, this study has some limitations that should be acknowledged. A major limitation is the lack of detailed information regarding age and sex of the control group, which mostly consisted of relatives of the Niemann–Pick disease type C participants. Additionally, the absence of neuropathological data is another limitation, as it restricts the ability to correlate clinical and biomarker findings with underlying brain pathology.

## Supplementary Material

fcae375_Supplementary_Data

## Data Availability

No new software and/or algorithms, in-house scripts or programmes were generated to support this study. Requests for the data sets used in the present study will be promptly reviewed by the corresponding authors and the University of Gothenburg to verify whether the request is subject to any intellectual property or confidentiality obligations. Anonymized data can be shared by request from any qualified investigator for the sole purpose of replicating procedures and results presented in the article, provided that data transfer is in agreement with EU legislation. Requests received will be reviewed by the Gothenburg University’s Committee to verify whether these are subject to any intellectual property or confidentiality obligations and compliance with ethical and data protection standards. All requests for code used for data analyses and data visualization will be promptly reviewed by the corresponding authors and the University of Gothenburg.
