# Supplementary material for: Plasma phosphorylated-tau217 is increased in Niemann–Pick disease type C
Source: Brain Commun. 2024 Oct 25;6(6):fcae375. doi: 10.1093/braincomms/fcae375 (PMC11535543; doi:10.1093/braincomms/fcae375)
Supplement: fcae375_Supplementary_Data [file fcae375_supplementary_data.docx]

**Supplementary material**

**Plasma phosphorylated-tau217 (p-tau217) is increased in Niemann-Pick disease type C**

Fernando Gonzalez-Ortiz, MD, MSc^1,2^; Thomas K. Karikari, PhD MSc^1,3^; Danielle Taylor-Te Vruchte^4^; Dawn Shepherd^4^; Bjørn-Eivind Kirsebom, PhD^5,6^; Tormod Fladby, MD, PhD^7,8^; Frances Platt, PhD^4^; Kaj Blennow, MD, PhD^1,2,9,10^

**Author affiliations:**

1. Department of Psychiatry and Neurochemistry, Institute of Neuroscience and Physiology, the Sahlgrenska Academy at the University of Gothenburg, Mölndal, Sweden
2. Clinical Neurochemistry Laboratory, Sahlgrenska University Hospital, Mölndal, Sweden
3. Department of Psychiatry, School of Medicine, University of Pittsburgh, Pittsburgh, PA 15203, USA
4. Department of Pharmacology, University of Oxford, Oxford OX1 3QT, UK
5. Department of Neurology, University Hospital of North Norway, Tromsø, Norway
6. Department of Psychology, Faculty of Health Sciences, The Arctic University of Norway, Tromsø, Norway
7. Department of Neurology, Akershus University Hospital, Lørenskog, Norway
8. Institute of Clinical Medicine, Campus Ahus, University of Oslo, Oslo, Norway
9. Institut du Cerveau et de la Moelle épinière (ICM), Pitié-Salpêtrière Hospital, Sorbonne Université, Paris, France
10. University of Science and Technology of China, Hefei, Anhui, P.R., Shenzhen, China

Correspondence to: Fernando Gonzalez-Ortiz

Address: Mölndal Hospital, Biskopsbogatan 27, SE-43180 Mölndal, Sweden

E-mail: Fernando.gonzalez.ortiz@gu.se

**Supplementary Methods**

**Statistical analyses**

Statistical analyses were performed with Prism version 9.3.1 (GraphPad, San Diego, CA, USA). Data are shown as mean ±standard deviation unless otherwise stated. The distributions of data sets were examined for normality using the Kolmogorov-Smirnov test. Non-parametric tests were used for non-normally distributed data. Spearman correlation and the χ2 test were used for continuous and categorical variables respectively. Group differences were examined using the Mann-Whitney test (two categories) or the Kruskal-Wallis test with Dunn’s multiple comparison (three or more groups).

**Data availability**

No new software and/or algorithms, in-house scripts or programs were generated to support this study. Requests for the datasets used in the present study will be promptly reviewed by the corresponding authors and the University of Gothenburg to verify whether the request is subject to any intellectual property or confidentiality obligations. Anonymized data can be shared by request from any qualified investigator for the sole purpose of replicating procedures and results presented in the article, provided that data transfer is in agreement with EU legislation. Requests received will be reviewed by the Gothenburg University’s Committee to verify whether these are subject to any intellectual property or confidentiality obligations and compliance with ethical and data protection standards. All requests for code used for data analyses and data visualization will be promptly reviewed by the corresponding authors and the University of Gothenburg.


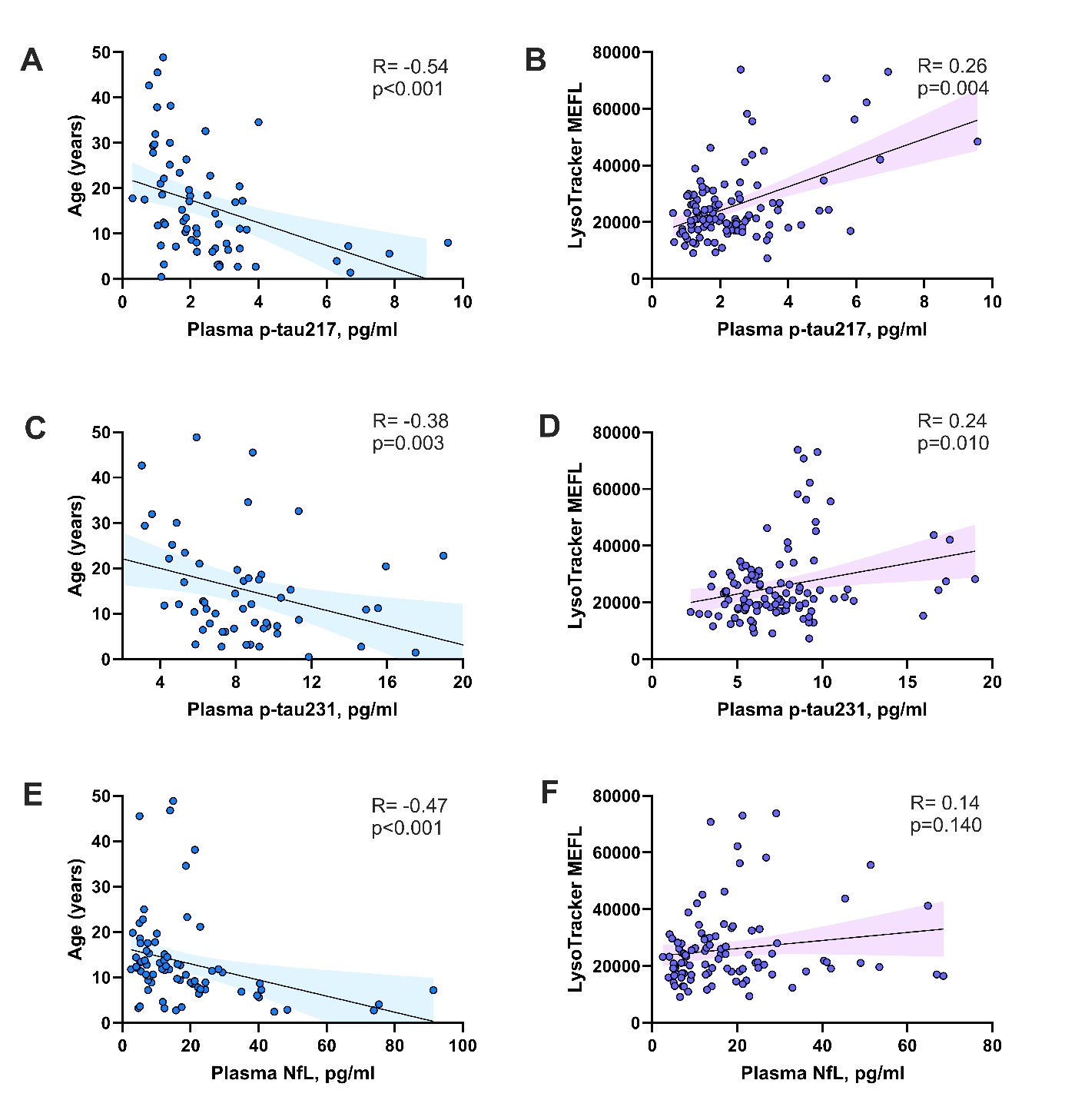


**Supplementary figure 1. Spearman correlation of plasma biomarkers with age at disease onset at baseline and mean equivalent of fluorescence (LysoTracker MEFL) longitudinally A)** Correlation between age of disease onset and levels of plasma p-tau217 at baseline. **B)** Correlation between longitudinal measurements of plasma p-tau217 and measurement of intracellular acidic LysoTracker MEFL. **C)** Correlation between age of disease onset and levels of plasma p-tau231 at baseline. **E)** Correlation between longitudinal measurements of plasma p-tau231 and MEFL/LysoTracker. **E)** Correlation between age of disease onset and levels of plasma NfL at baseline. **F)** Correlation between longitudinal measurements of plasma NfL and MEFL/LysoTracker. The colored bands are 95% confidence intervals.


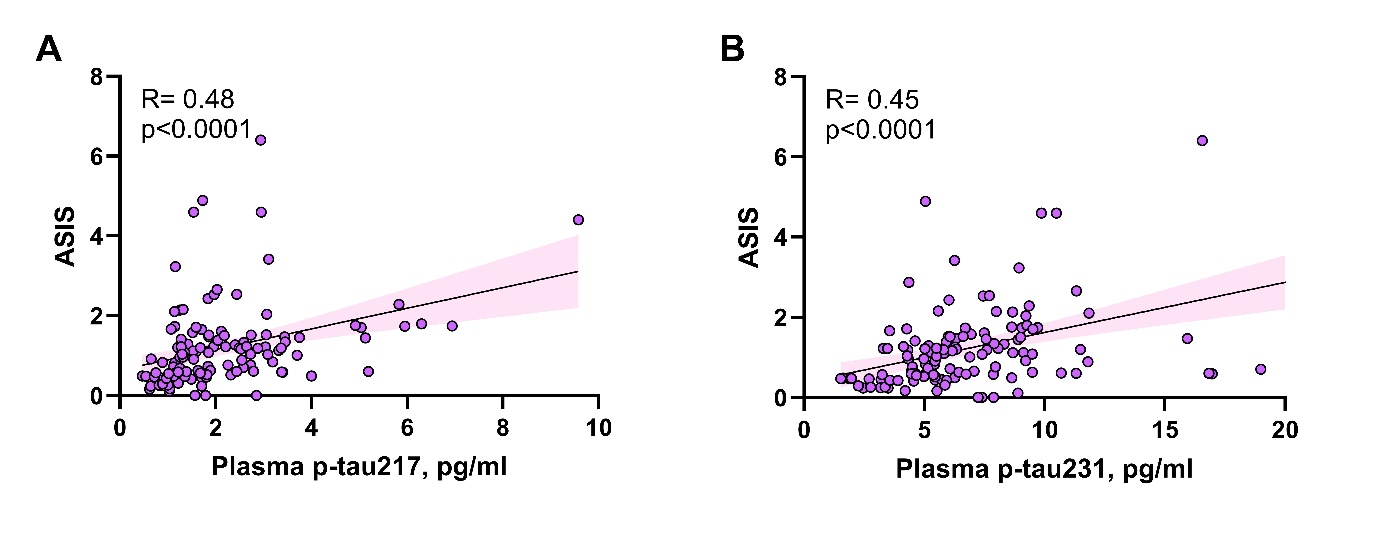


**Supplementary figure 2. Spearman correlation of longitudinal measurements of plasma p-tau biomarkers with Annual Severity Increment Score (ASIS). A)** Correlation between longitudinal measurements of plasma p-tau217 and ASIS. **B)** Correlation between longitudinal measurements of plasma p-tau231 and ASIS. The colored bands are 95% confidence intervals.

**Supplementary table 1**.- Demographic information of controls and NPC patients

| **Variable** | **HC (N=60)** | **NPC (N=71)** | **p-value** |
| --- | --- | --- | --- |
| **Age** | N/A | 16.01 (+/- 11.65) | N/A |
| **Females (%)** | N/A | 39/71 (54.9%) | N/A |
| **Plasma p-tau217 (pg/ml)** | 1.02 (+/- 0.34) | 2.52 (+/- 1.93) | <0.001 |
| **Plasma p-tau231 (pg/ml)** | 5.51 (+/- 2.40) | 8.70 (+/- 3.85) | <0.001 |
| **Plasma NfL (pg/ml)** | 6.34 (+/- 4.19) | 21.73 (+/- 17.70) | <0.001 |

Abbreviations. HC, healthy controls; NPC, Niemann Pick disease type C; p-tau, phosphorylated tau; NfL, Neurofilament Light Chain; N/A, not available. Data are shown as mean ± standard deviation. P values indicate the results of Mann-Whitney tests. Detailed information regarding age and sex of the control group, which mostly consisted of relatives of the NPC participants, was not collected.

**Supplementary table 2.-** Demographic information of the AD group

| **Variable** | **Overall, (N=60)** | **A+T- AD, (N=30)** | **A+T+ AD, (N=30)** | **p-value** |
| --- | --- | --- | --- | --- |
| **Age** | 66.21 (7.41) | 68.23 (8.04) | 65.60 (7.34) | 0.2 |
| **Females (%)** | 39 / 60 (65%) | 18 / 30 (60%) | 21 / 30 (70%) | 0.4 |
| **Plasma p-tau217 (pg/ml)** | 2.97 (+/-1.30) | 2.67 (+/-1.18) | 3.26 (+/-1.3) | 0.09 |

Abbreviations*.* A+/-, positive or negative Cerebrospinal Fluid (CSF) marker for amyloid plaques; T+/-, positive or negative CSF marker for p-tau; p-tau. Data are shown as mean ± standard deviation. P values indicate the results of Mann-Whitney tests.
